# Making Use of Construction Waste in Soil-Cement Mixtures: Granulometric Correction of Clay Soil

**DOI:** 10.1021/acsomega.4c10911

**Published:** 2025-08-22

**Authors:** Jéssica Filipe Briskievicz, Carolina Angulski da Luz, Fernanda Batista de Souza

**Affiliations:** † Universidade Tecnológica Federal do Paraná (UTFPR), Programa de Pós- graduação em Engenharia Ambiental Análise e Tecnologia Ambiental (PPGEA), Câmpus Francisco Beltrão, Rua Gelindo João Folador, n°2000, PO Box 135, Francisco Beltrão, Paraná CEP 85602-863, Brazil; ‡ Universidade Tecnológica Federal do Paraná (UTFPR), Departamento de Engenharia Civil, Câmpus Pato Branco,Via do Conhecimento, km 1, Pato Branco, Paraná CEP 85503-390, Brazil

## Abstract

Construction is fundamental to human development, but it is the sector of activity that uses the most natural resources and produces around 50% of the solid waste generated by human activity. In this research, mixed recycled aggregate (MRA) with the granulometry of small aggregate was used for the physical-mechanical investigation of different mixtures of soil-cement and MRA. The granulometry of the soil and the MRA were characterized, followed by a two-level factorial experimental design complete with a central point. The ratio of MRA to soil was 1.00, 1.67, and 2.33. The cement content added to the mixture varied between 10% and 14%. The best result was obtained by incorporating 60.2% MRA with 25.8% soil (MRA/soil of 2.33) and 14% cement (mass % of solids), achieving a compressive strength of 3.4 MPa and water absorption of 18.1%. The prediction of the values for the MRA/soil and % cement factors considering the minimum compressive strength of 2.1 MPa by the multiple regression model indicated a mixture with an MRA/soil ratio of 1.71 and 10.22% cement. This mixture indicated a predictable maximum water absorption of less than 22%. The predictability results are promising as they allow the incorporation of CDW in the manufacture of soil-cement blocks to be scaled up, increasing its recyclability. This highlights the need to correct clay soils and the potential for recycling of construction and demolition waste.

## Graphical abstract

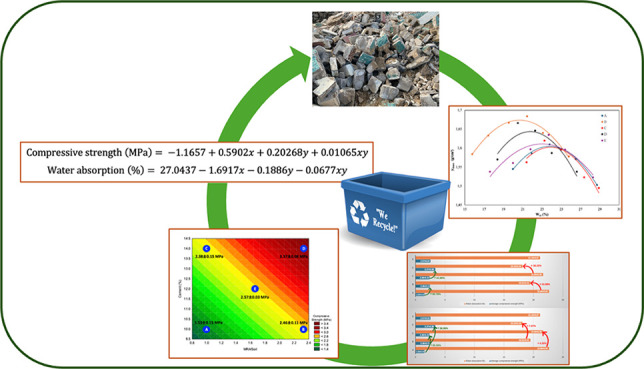

## 1 Introduction

Construction has a negative impact on the environment, ranging from the extraction of raw materials to the generation of solid waste.^1^ One way to reduce this impact is by incorporating construction and demolition waste (CDW) into manufacturing new materials, such as soil-cement mixtures.^2^ Soil-cement for manufacturing bricks is considered an environmentally friendly material compared to conventional bricks. The use of clay soils in soil-cement mixes presents several challenges, mainly due to their compressibility and low strength properties; they are prone to significant changes in volume with variations in moisture, leading to instability in structures built on them. Additionally, they have high plasticity, which can result in excessive deformation under load. These characteristics can negatively affect the quality of construction.

Particle size correction is essential to mitigate these problems and improve the performance of the mixtures. Adjusting the granulometric composition helps to obtain a more stable soil matrix, improving compaction and reducing voids, which leads to better compressive strength values.

Using waste to amend clay soils promotes sustainability and sparks interest in reducing carbon emissions compared to more conventional materials, which require the development and innovation of alternative materials and techniques to meet the structural requirements of construction.^3^

Soil-cement is a building material made up of soil, cement, and water. Additives or pigments can be added as long as they do not interfere with the properties required by the standards. Investigating each component’s influence on soil-cement’s physical properties requires extensive testing. In addition, evaluating the interactive effects between these components using the controlled variable method presents significant challenges. Consequently, determining the optimal proportions of each element in the mixture requires a methodical approach to experimental design.^4^

Soil is the primary raw material for soil-cement. It needs to have characteristics that allow it to meet quality requirements and minimize the use of cement, to lack organic matter in quantities that could affect the hydration of the cement and the stabilization of the soil, and to exhibit good workability in its fresh state. The granulometric composition of the soil varies between sand, silt, and clay. Motta et al.^5^ indicate that the soil used in soil-cement production should have a sand content of between 50% and 70%, a silt fraction of between 10% and 20%, and a clay fraction of 10% and 20%. Sandy soils are recommended because they need less cement to be stabilized, while clayey soils require correction.

The production of soil-cement materials is gaining prominence in sustainability, as they do not use the firing process in their manufacture. Manufacturing these materials can require less than 10% of the energy needed to make burnt clay bricks and concrete masonry units. Soil-cement materials are produced by the homogeneous compression of a mixture of soil, water, and a stabilizing material (Portland cement) that gives the product high strength and durability.^3^

Stabilized soil blocks, such as soil-cement, are considered low-carbon and low-embodied-energy materials, as they do not require the firing/baking of the blocks and can save up to 60–70% of energy compared to fired clay bricks. They can be molded into any shape and size required and designed for the strength needed by varying the stabilizer content.^6^ Using soil-cement is common in paving works and the construction of small- and medium-sized residential buildings, replacing bricks and ceramic blocks.^7–9^

Several studies have been carried out on the incorporation of different types of waste in the manufacture of eco-efficient bricks, such as sugarcane bagasse ash,^10^ sawdust, paper fiber, manure effluent,^11^ and construction waste,^12^ to improve the characteristics of soil-cement products or replace the binder used, as well as to allow the correction of clayey soils. Table 1 summarizes the results obtained from studies incorporating different types of waste in the production of soil-stabilized bricks, focusing on compressive strength (28 days of curing) and water absorption (7 or 28 days of curing).

**Table 1:** Incorporation of Waste in the Production of Soil-Stabilized Bricks^a^

Waste	Dosage (%)	Water absorption (%)	Compressive strength (MPa)	Reference
Silica fume	5–20	16.52–17.32	23	^13^
Waste from the iron ore extraction process	1:9 (cement:soil) With 0, 10, 20, 30, and 40% replacement of the soil with residue	15.5–17.0	2.66–3.38	^3^
Ceramics	2 and 4 in soil-cement-lime mixtures	15.7–16.7	1.45–1.60	^14^
Composite of ash, sawdust, paper fiber, manure effluent	(clay:sand:ash:sawdust:paper fiber) (80:10:8:1:1); (75:12:10:2:1) e (70:13:12:3:2)	3.16–4.19	0.92–1.03	^11^
Pisha sandstone	0–9 with 3–12% cement	nd^b^	1.38–5.83	^4^
CDW	25 and 50 with 12 cement	11.15–15.88	0.61–1.85	^12^
CDW	75 and 100 with 8 and 7 binder	15.19–15.86	Average of 2.5	^15^
CDW	25–75 with 7 cement	nd^b^	1.18–2.82	^16^
CDW	60–100 with 6 cement	15.4–16.1	4.6–5.1	^17^

a nd* - not done.

b Compressive strength was determined at the international standard of 28 days postmixing.

To evaluate the potential of correcting clay soils with mixed recycled aggregate (MRA) from CDW in obtaining soil-cement mixtures, this work proposes a statistical evaluation of the influence of the MRA/soil ratio and the percentage of cement (binder) on the compressive strength and water absorption in cylindrical specimens.

## 2 Materials and Methods

### 2.1 Characterization of Materials

MRA was used to evaluate the processing of waste at the recycling plant. MRA contains less than 90% by mass of Portland cement-based fragments and rocks and is brown in color.

The average specific mass of MRA is 2.34 ± 0.02 g/cm^3^. The particle size analysis shows that the aggregate has a smooth, elongated horizontal distribution, indicating a well-graded continuous particle size. With 17% of the material passing the 0.075 mm sieve, the aggregate is classified as fine sand due to its fineness modulus (MF) of 1.64, which is less than 2.4. The characteristic maximum dimension of the aggregate is 2.36 mm.

The chosen binder was Portland cement CPII-Z from the Itambé brand because it is recommended in the technical bulletin of the Brazilian Portland Cement Association (ABCP),^18^ a publication that aims to disseminate standard techniques for applying soil-cement in housing construction. Further details on the characterization of MRA and the binder are presented in Supporting Information.

The soil was collected in the city of Pato Branco, Paraná, Brazil, where the soil was turned over based on the work of Briskievicz,^19^ Beutler,^20^ and Vieira^21^ for the manufacture of soil-cement products. The soil collected was classified as (LVdf1) Latossolo Vermelho Distroférrico according to the Brazilian Soil Classification System (SiBCS).

The soil and MRA/soil mixtures were prepared and characterized by following the Brazilian Regulatory Standards (NBR) of the Brazilian Association of Technical Standards (ABNT). Soil preparation was carried out according to NBR 6457.^22^ For the liquidity limit test, NBR 6459 ^23^ was used, and for the plasticity limit, NBR 7180.^24^ The granulometric analysis by sedimentation was carried out according to the Brazilian Agricultural Research Corporation (Embrapa) methodology.^25^

The characterization of the soil amended with MRA was carried out to check that the granulometric composition complied with the values established in the ABCP Technical Bulletin 117 ^18^ and NBR 11798.^26^

A soil granulometry correction method was used to check whether the corrected soil complied with the three compositions required for soil-cement production. To do this, the classification of NBR 6502 ^27^ was considered, in which the clay and silt fraction of the soil have grains smaller than 0.06 mm, and the sand fraction has grains larger than 0.06 mm. The granulometry of the CDW MRA was considered from the 4.75 mm sieve to the fraction that passes through the 0.075 mm sieve since this fraction is also part of the composition of the corrected soil.

To adjust the soil, 1000 g of the mixture was prepared, and the required material amount was determined for each composition. Based on the results, the quantities of sand, silt, and clay for each composition were estimated.

The liquidity limit (LL) and plasticity index (PI) tests were carried out to assess the compliance of the corrected soil with the soil classification in NBR 11798.^26^ The tests followed standards NBR 6459 ^23^ and NBR 7180 ^24^ for the liquidity and plasticity limits, respectively. They were repeated twice for each soil sample corrected with MRA at different moisture contents and for all soil correction dosages. Further details on the natural and amended soil characterization are given in Supporting Information.

### 2.2 Dosage

Considering that the soil in the region studied is predominantly clay, it was necessary to correct it so that it possessed the characteristics of sandy soil, which are essential for the manufacture of soil-cement. To make this correction, recycled aggregate from construction waste (MRA) with grain diameters between 0.05 mm and 4.8 mm was chosen and used in different proportions with clay soil.

Based on Technical Bulletin 117 from ABCP,^18^ which indicates sand contents of between 50% and 90% and silt and clay between 10% and 50%, and taking into account the need for sufficient initial strength for handling and removing the specimens from the mold, three compositions were established: 50% soil and 50% MRA (ratio 1.0), 30% soil and 70% MRA (ratio 2.33), and a central composition with 37.5% soil and 62.5% aggregate (ratio 1.67).

Three cement contents were selected, with the lowest point limited to 10%, the highest point limited to 14%, and the central point set at 12%. These cement dosages refer to the total mass of the mixture, as shown in Table 2.

**Table 2:** Factorial Experimental Design for % Cement and MRA/Soil Ratio in Soil-Cement Dosage^a^

Level	Cement	Soil + MRA	MRA/soil ratio
–1	10%	90%	1.00 (50% soil/50%MRA)
0	12%	88%	1.67 (62.5% MRA/37.5% soil)
+1	14%	86%	2.33 (70% MRA/30% soil/)

a The lowest level of the factors is coded as −1, the central level is coded as 0, and finally, the highest level is coded as +1 in the mathematical and statistical treatment of the data.

The cement content in the mixture and the MRA/soil ratio were chosen based on previous studies.^12,14–17,19^

The experimental design included five different combinations of corrected soil and cement, as shown in Table 3. Two 2^2^ experimental designs were evaluated: one for the dependent variable compressive strength (triplicate) and the other for water absorption (duplicate). A 95% confidence level was adopted for the statistical analyses, and the *p*-value was set at 0.05 for the variance analysis (ANOVA). The regression model’s determination coefficient (*R* ^2^)^29^ was calculated, and the response surfaces for the dependent variables were obtained using the software TIBCO Statistica.

**Table 3:** Nomenclature Provided for Samples According to Cement and MRA/Soil Ratio Dosages

Sample	Cement	MRA/soil ratio
A	10%	1.00
B	10%	2.33
C	14%	1.00
D	14%	2.33
E	12%	1.67

The samples were molded following the guidelines for method A of standards NBR 12024 ^30^ and NBR 12023.^31^ A homogeneous mixture of corrected soil and cement was obtained, and water was added in an adequate quantity to reach the optimum humidity previously determined for each dosage, also taking into account the loss of water through evaporation, with an increase of 0.5 to 1.0% points of moisture. Mixing was continued until the materials were completely homogenized.

Figure 1a shows the equipment used for the normal Proctor compaction tests and the molding of cylindrical specimens. In addition, a humidity chamber was used with a relative humidity of no less than 95% and a temperature of 23 °C with a variation of ±2 °C, for which plastic containers with lids were used along with a sheet of water to provide the humid curing indicated by the standard for 7 days.

**Figure 1:**
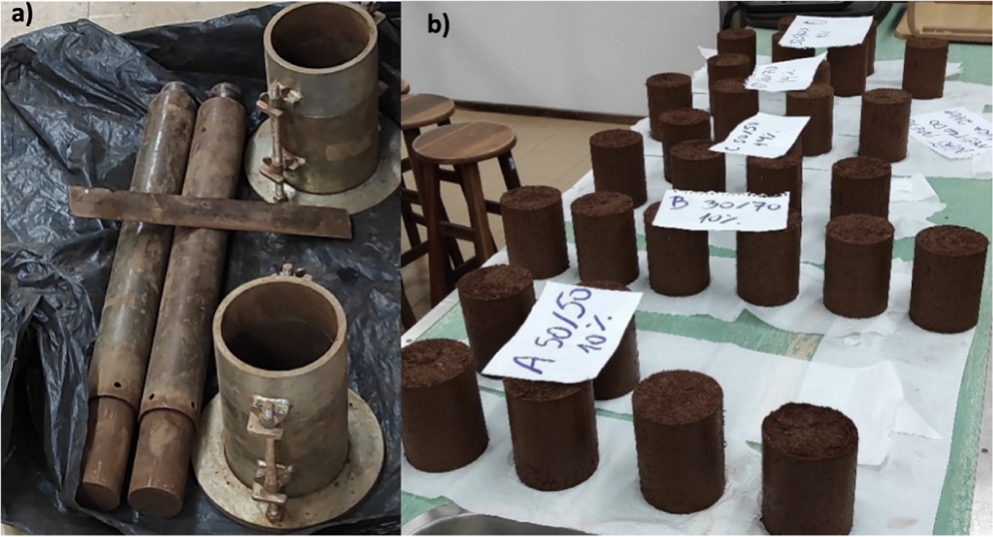
(a) Equipment used for molding cylindrical samples with a diameter of 100.0 ± 0.4 cm and a height of 127.3 ± 0.3 cm and for normal Proctor compaction tests. (b) Samples molded by NBR 12024 ^30^ during the curing process, separated according to the nomenclature presented in Table 3. Source: Adapted with permission from BRISKIEVICZ, J. F. Valorização de agregado de resíduos da construção civil na correção granulométrica de solos argilosos para produção de solo-cimento. 2022. Federal Technological University of Paraná, [s.l.], 2022. Licensed under Creative Commons CC BY-NC-SA.

For all dosages, the degree of compaction was checked following NBR 12024,^30^ which establishes the methods for molding and curing cylindrical soil-cement specimens. The dimensions of the specimens are 127.3 ± 0.3 cm in height and 100.0 ± 0.4 cm in diameter, with a mass of around 5 kg per sample. A total of 25 cylindrical soil-cement specimens were molded (Figure 1b) according to the dosages defined by the experimental design: 15 samples for the compressive strength test and 10 samples for the water absorption test.

### 2.3 Mechanical Performance Tests

The performance tests were carried out following the procedures described in NBR 12025 ^32^ for determining the simple compressive strength (Figure 2a) and NBR 13555 ^33^ for the water absorption capacity (Figure 2b) of cylindrical soil-cement specimens. The tests were carried out at 28 days of age.

**Figure 2:**
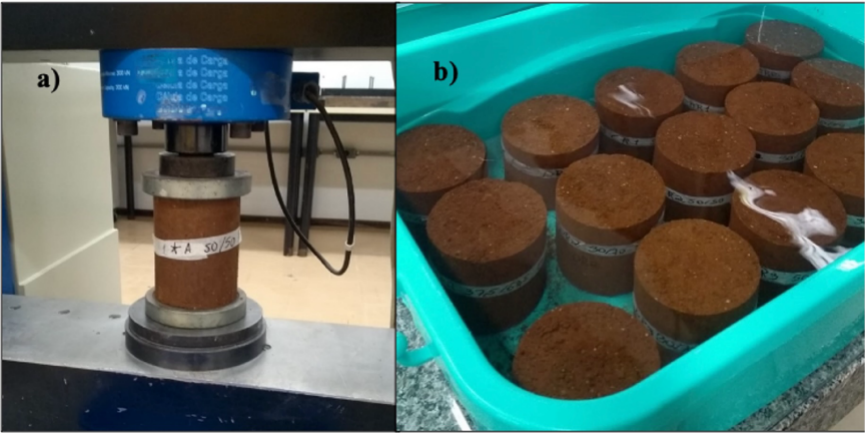
Samples during mechanical performance tests: (a) Compression strength test in Proctor cylinder equipment with 1 mm/min controlled deformation. The maximum load achieved was recorded as the breaking load of the test specimen with a resolution of 50 N. (b) Soil-cement samples were immersed in water in a humid chamber for 24 h, at 25 °C. After this period, each test specimen was weighed to determine its wet mass. Source: Adapted with permission from BRISKIEVICZ, J. F. Valorização de agregado de resíduos da construção civil na correção granulométrica de solos argilosos para produção de solo-cimento. 2022. Federal Technological University of Paraná, [s.l.], 2022. Licensed by Creative Commons CC BY-NC-SA.

## 3 Results and Discussion

### 3.1 Soil Characterization

The results of the soil characterization tests and the reference values are presented in Table 4. According to standard NBR 11798,^26^ soils considered suitable for soil-cement production belong to classes A1, A2, or A4, according to the ASTM D3282 ^34^ classification. However, the soil under study was classified as A-7, a class commonly associated with materials with low bearing capacity and high fines content.

**Table 4:** Characterization of the Soil Used in the Manufacture of Cylindrical Soil-Cement Test Specimens before Granulometric Correction^a^

Test	Results	Reference ASTM D3282 and NBR 11798	
Silt+clay content (through sieve n° 200)	94.4%	Min 36%	A-7
Liquid limit (LL)	59 ± 1.87%	Min 41%	
Plasticity index (PI)	17 ± 2.44%	Min 11%	
Sieve passage 75 mm	100%	100%	
Retained on the 19 mm sieve	0%	Max 30%	
Retained on the 4.75 mm sieve	0%	Max 40%	
Sand content	5.6%	50% to 90%	
Silt + clay content	94.4%	10% to 50%	

a Source: Adapted by the author based on refs 18,34,35 .

When analyzing the granulometric composition, it is observed that the soil has a silt + clay content of 94.4%, a value well above the recommended range of 10% to 50% and considerably higher than the minimum value of 36% required for class A-7. In contrast, the sand content is only 5.6%, below the ideal range of 50% to 90%, reinforcing the predominance of fine particles in the material.

The results for the liquidity limit (LL) and plasticity index (PI), with values of 59 ± 1.87% and 17 ± 2.44%, respectively, are above the minimum values required by the standard for classification A-7 (LL ≥ 41% and PI ≥ 11%). These values indicate a soil with high plasticity, which can impair the workability and volumetric stability of the soil-cement mixture.

In addition, the soil passed entirely through the 75 mm sieve and was not retained in the 19 and 4.75 mm sieves, meeting the granulometric requirements for maximum grain size. However, the unbalanced distribution between sand, silt, and clay, with an excess of fine particles, compromises the soil’s performance as a matrix for cement stabilization.

Thus, although the soil meets some technical criteria, the results show that adjustments to the granulometric composition are essential. Adding granular materials, such as MRA, may be necessary to reduce the fines content and improve the compactness, strength, and durability characteristics of the soil-cement mixture.

### 3.2 Characterization of Soil Amended with MRA from CDW

The fraction corresponding to silt plus clay in the soil was obtained by adding up the results of the granulometric analysis by sedimentation, which indicated 16.4% silt and 78% clay, respectively, giving a total of 94.4% silt plus clay. The sand fraction of the soil was determined to be 5.6%. With this profile, the soil is likely to be found in the region of clayey or silty-clay soils, indicating high plasticity and low drainage—undesirable characteristics for direct use in soil-cement. The MRA of CDW was composed of 100% sand and had no silt or clay content (Table 5).

**Table 5:** Granulometric Composition of Soil and MRA in Terms of Sand and Silt + Clay Content before Correction

Materials	Sand	Silt + clay
Soil	5.6%	94.4%
MRA	100%	0%

Using a proportion, the percentages of sand, silt, and clay present in each of the amended soil formulations were obtained, as shown in Table 6.

**Table 6:** Soil Composition Corrected with MRA for Sand and Silt + Clay Content

Materials	Sand	Silt + clay
50% soil + 50% MRA	52.8%	47.2%
37.5% soil + 62.5% MRA	64.6%	35.4%
30% soil + 70% MRA	71.7%	28.3%
Reference values^a^	50% to 90%	10% to 50%

a Reference based on Technical Bulletin 117 from ABCP.^18^

The initial characterization of the soil revealed a strongly clayey texture, with 78.0% clay, 16.4% silt, and only 5.6% sand, corresponding to a very clayey soil classification according to the SiBCS (Sistema Brasileiro de Classificação de Solos - Brazilian Soil Classification System) texture triangle.^39^ This composition is in line with class A-7 of the ASTM D3282 classification, which is generally unsuitable for direct use in soil-cement production due to its high fine content and low granular fraction. Soils with these characteristics tend to have high plasticity, high water retention capacity, compaction difficulties, and greater susceptibility to volumetric shrinkage during curing.^26,34^

To adapt the soil to the requirements of technical standards, granulometric corrections were made by adding MRA in three different proportions: 50%, 62.5%, and 70%. Figure 3 summarizes the resulting granulometric compositions after each correction.

**Figure 3:**
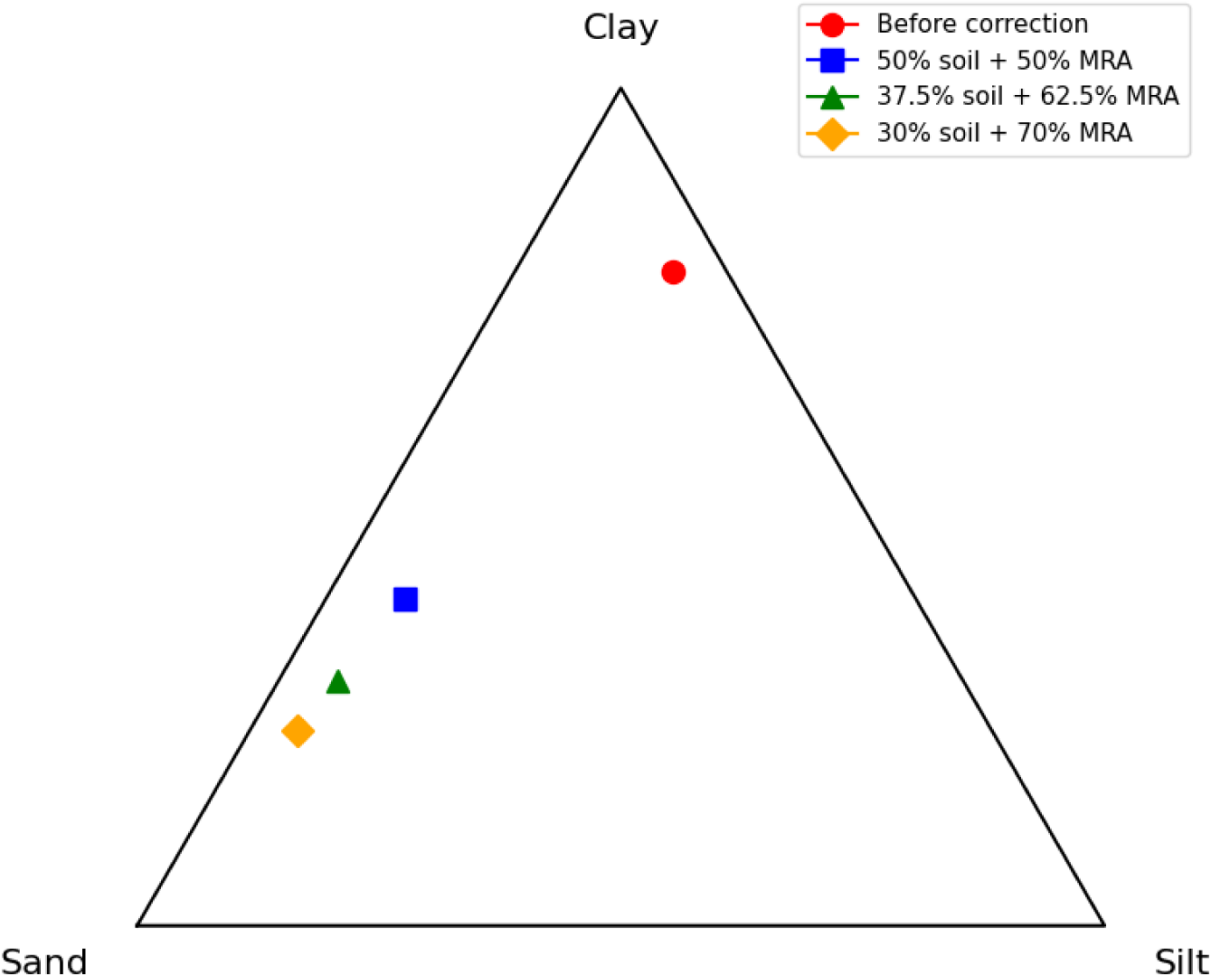
Soil texture triangle before and after granulometric corrections.

The progressive addition of MRA promoted a substantial redistribution of the textural fractions, with a systematic reduction in the clay content and an increase in the sand fraction. The mixture with 50% MRA already significantly improved the texture, with characteristics potentially more suitable for compaction and cement incorporation, whose classification is sandy loam soil.^39^ The proportion of 64.6% sand in the correction with 62.5% MRA and 71.7% sand in the correction with 70% MRA resulted in soils with clay contents between 29.3% and 23.4%, approaching the recommended ranges for stabilization with cement, whose soil classification is sandy loam.^39^

The main objective of granulometric correction is to balance the soil texture, aiming to optimize the compactability, mechanical resistance, and durability of the soil-cement mixture. The reduction in clay content contributes to less shrinkage during curing and greater adhesion between the soil and the cement hydration products. At the same time, the increase in the granular fraction (sand) favors particle packing and stress distribution.

Finally, Table 7 shows the results of the characterization of the corrected soil for the three dosages, together with the reference values from NBR 11798 ^26^ and Technical Bulletin 117,^18^ showing that the corrected soil with the dosages of 50% soil and 50% recycled construction aggregate (ratio 1.0), 30% soil and 70% recycled construction aggregate (ratio 2.33), and 37.5% soil and 62.5% aggregate (ratio 1.67) meets the requirements to be used in the production of soil-cement.

**Table 7:** Characterization and Classification of Soil Corrected with MRA Used in Soil-Cement Production^a^

	Results			
Test	50% solo + 50% MRA	37.5% solo + 62.5% MRA	30% solo + 70% MRA	Reference ASTM D3282 and NBR 11798
Silt+clay content (through sieve n° 200)	47.2%	35.4%	28.3%	A-1, A-2, or A-4
Liquid limit (LL)	34 ± 0.29%	29 ± 0.06%	28 ± 1.31%	
Plasticity index (PI)	9 ± 1.22%	4 ± 0.06%	3 ± 0.83%	
Sieve passage 75 mm	100%	100%	100%	100%
Retained on the 19 mm sieve	0%	0%	0%	Max 30%
Retained on the 4.75 mm sieve	0%	0%	0%	Max 40%
Sand content	52.8%	64.6%	71.7%	50% to 90%
Silt + clay content	47.2%	35.4%	28.3%	10% to 50%
ASTM D3282 soil classification	A-4	A-2–4	A-2–4	-

a Source: Adapted by the author based on refs 18,34,35 .

The granulometric correction of clay soils significantly influences soil-cement production by improving the material’s properties, making it more suitable for construction applications. This correction involves adjusting the particle size distribution of the soil, which can enhance its compaction, strength, and overall performance in soil-cement mixes.

### 3.3 Compaction Characteristics

Figure 4 shows the compaction curves for each sample, and Table 8 shows the equation for each curve and the values for optimum moisture (*W*_ot_) and maximum dry specific mass (γ_dmax_).

**Figure 4:**
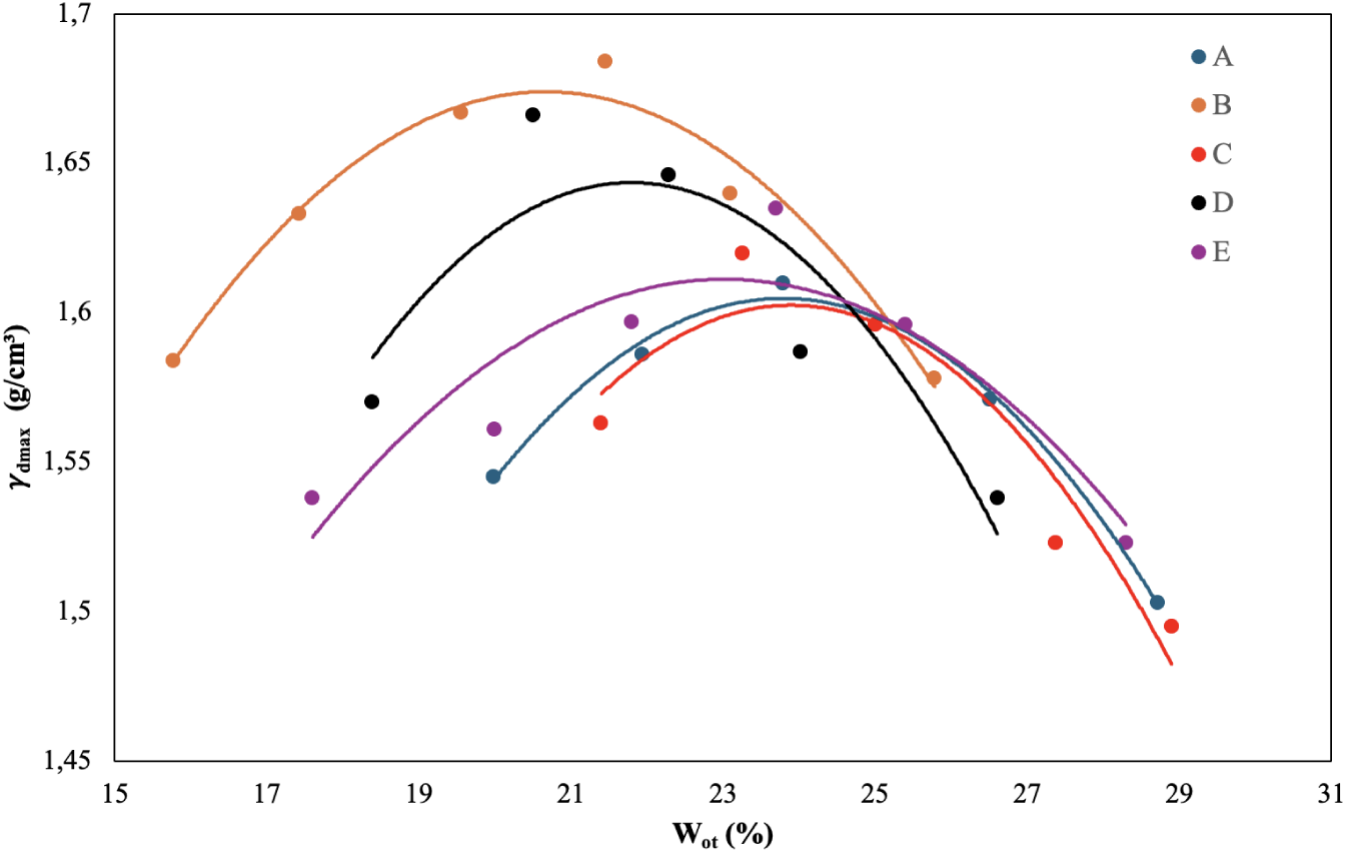
Compaction curves for each composition based on the ratio of maximum dry specific mass (γ_dmax_) to optimum average moisture (*W*_ot_).

**Table 8:** Optimal Average Moisture (*W*_ot_) and Maximum Dry Specific Mass (γ_dmax_) of Cylindrical Soil-Cement Blocks Obtained from Parabolic Models of Compaction Curves

Sample	Cement (%)	MRA/soil ratio	Equation	*W*_ot_ (%)	γ_dmax_ (g/cm^3^)
A	10	1.00	*y* = −0.0042*x* ^2^ + 0.2004*x* – 0.7791	23.86	1.611
B	10	2.33	*y* = −0.0038*x* ^2^ + 0.1558*x* + 0.0636	20.50	1.661
C	14	1.00	*y* = −0.0048*x* ^2^ + 0.2286*x* – 1.1279	23.81	1.594
D	14	2.33	*y* = −0.0051*x* ^2^ + 0.2207*x* – 0.7617	21.63	1.626
E	12	1.67	*y* = −0.0029*x* ^2^ + 0.1353*x* + 0.0540	23.33	1.632

The optimum moisture content for dosage A was 23.86%, for B 20.50%, for C 23.81%, for D 21.63%, and for E 23.33% (Table 8). The dosage of 50/50 corrected soil had the highest optimum moisture content, while the samples with the lowest optimum moisture content were those with 30/70 corrected soil. In addition, the optimum moisture content’s intermediate value corresponded to the dosage’s central point, which consisted of 37.5/62.5 of amended soil. The variations in cement dosages do not influence the optimum moisture content. As for the maximum dry apparent density, for the 10% cement dosage, there is a 3.1% increase in the γ_dmax_ with the increase in the fraction of MRA in the composition, while for the 14% cement dosage, the increase in the γ_dmax_ is 2%. Vilela et al.^3^ also observed a decrease in optimum humidity and an increase in maximum dry apparent density with an increase in the waste fraction in the soil-cement composition.

The compaction curves shown in Figure 3 have a parabolic shape as per ABNT standard NBR 12023.^31^ Therefore, the polynomial model of order two was adjusted to the experimental data to obtain the equations that describe the compaction curve (Table 8). The coefficients of determination (*R* ^2^) of the adjustments to the parabolic model ranged from 0.7958 (sample D) to 0.9918 (sample A).

This result is due to the density of MRA, which is 2.34 ± 0.02 g/cm^3^, as well as its particle size distribution, in which 17% of MRA passes the 0.075 mm sieve, its characteristic maximum dimension is 2.36 mm, and its fineness modulus (MF) is 1.64. These characteristics allow MRA to act as a filler in the mix, reducing pores and facilitating compaction. This results in denser and more resistant soil-cement materials with lower water absorption and excellent durability.^3^

The improved particle size distribution obtained from the MRA addition reshaped the particle size curve of the clay soil, making it resemble sandy soils, which improves its texture and reduces plasticity (see Supporting Information).

### 3.4 Statistical Analysis of Mechanical Performance

Figure 5 shows the average results for compressive strength and water absorption. The only dosage that did not reach the minimum strength of 2.1 MPa at 28 days was the MRA/soil ratio of 50/50 with 10% cement, which obtained an average compressive strength of 1.53 ± 0.15 MPa. This dosage exceeded the maximum water absorption limit of 22%, registering 22.66 ± 0.04%. By increasing the cement dosage to 14% while maintaining the 50/50 MRA/soil ratio, there was an increase in compressive strength of 55.55% and a reduction of 4.50% in water absorption, allowing the dosage to meet the values required by the standards. The results obtained from the central point, with a ratio of 62.5/37.5 MRA/soil and 12% cement, showed an average compressive strength of 2.57 ± 0.03 MPa and water absorption of 21.13%. Similarly, the dosages with a 70/30 MRA/soil ratio and 10% cement content showed a compressive strength of 2.46 ± 0.11 MPa and water absorption of 19.51 ± 0.41%. In contrast, for the 14% cement content, the compressive strength increased by 36.99%, and the water absorption decreased by 7.07%.

**Figure 5:**
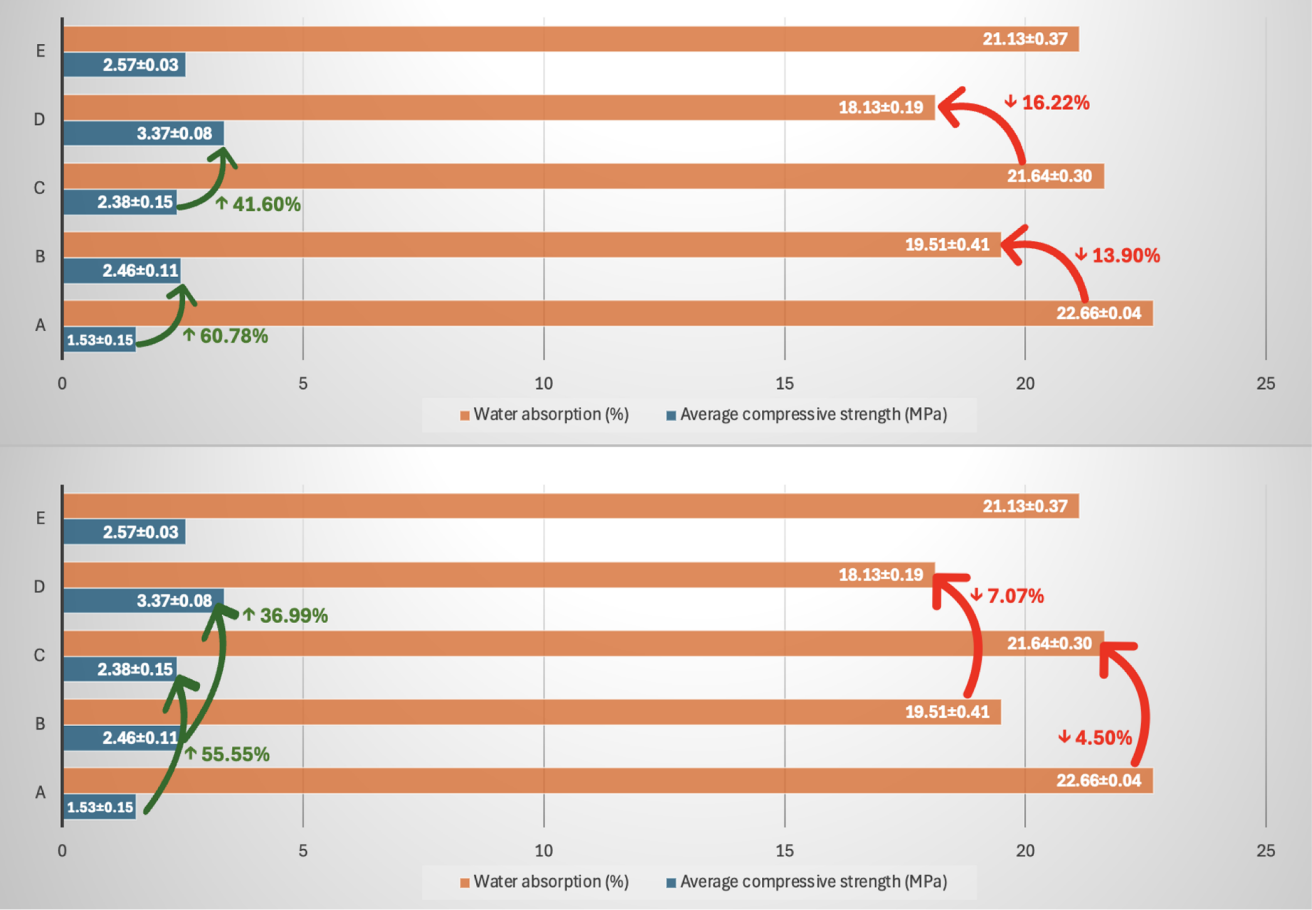
Compressive strength (MPa) and water absorption (%) for cylindrical soil-cement blocks. The green arrows indicate the increase in compressive strength (%), while the red arrows indicate the reduction in water absorption (%) when comparing the samples.

Figure 5 also shows that the MRA/soil ratio variation influences the properties studied while maintaining the binder dosages. For the 10% cement dosage, increasing the amount of MRA residue in the composition led to the most significant increase in compressive strength, which was 60.78%. For 14% cement, the increase in compressive strength was 41.60%. Regarding the water absorption results, there was a decrease of 13.90% (10% cement) and 16.22% (14% cement) with an increase in the amount of MRA waste in the composition. Stabilizing clay soils with cement leads to an increase in compressive strength, especially when the water/cement ratio of the soil is optimized. This is fundamental to achieving the desired engineering properties in construction.

The results showed that increasing the MRA/soil ratio and the cement dosage in the mix resulted in adequate results for compressive strength and water absorption according to the standards studied, but increasing the MRA/soil ratio had a more significant impact. The dosage with a ratio of 70/30 corrected soil and 14% cement showed the best results in compressive strength (3.37 ± 0.08 MPa) and water absorption (18.13%), meeting the limits set by the standards. The increase in compressive strength is due to different interactions. These interactions mainly involve the chemical and physical properties of the soil-cement matrix, influenced by factors such as cement hydration and particle size distribution. Cement hydration increases the strength through the formation of hydrated crystals and the agglomeration of soil particles. About soil granulometry, it is known that the gradation of the soil affects the compaction density and the void ratio. Incorporating MRA reduces porosity and improves mechanical response, thereby increasing the soil-cement’s compressive strength.

Ferrari et al.^10^ obtained results for the average strength in simple compression for hollow soil-cement bricks aged 21 days, ranging from 1.19 to 3.18 MPa, with compositions containing 6% to 9% cement and the addition of ash ranging from 0% to 20% of cement. The increase in ash content in the composition reduced the compressive strength. The compressive strength values obtained with the incorporation of CDW into soil-cement by Silveira and Nóbrega^15^ and Silva and Lafayatte^16^ corroborate those found in this research. Segantini and Wada^17^ reported better results for compressive strength (>4.6 MPa) and water absorption (<15.4%) with the addition of CDW in the soil-cement formulation, evaluating the incorporation of 60 to 100% CDW with 6% cement.

Table 9 shows the analysis of variance (ANOVA) for the variable compressive strength as a function of cement dosage and the ratio of soil to MRA. The interaction between the independent variables is insignificant; therefore, only the individual effects of these variables had a statistically significant influence on compressive strength. The determination coefficient (*R* ^2^) is 0.9665, indicating that around 96.65% of the variation in compressive strength is explained by the variables analyzed; i.e., the difference in compressive strength results is due to the dosages applied to correct the soil granulometry (MRA/soil ratio) and the amount of cement in the mixture. Only 3.35% of the variability in compressive strength is due to other factors not covered in the study.

**Table 9:** Analysis of Variance (ANOVA) for Compressive Strength and Water Absorption

Compressive strength					
Factors	Sum of squares	Degree of freedom	Mean square	F-calculated	*p*-value
(1) MRA/soil	2.736075	1	2.736075	171.2496	0.000000
(2) Cement	2.332008	1	2.332008	145.9592	0.000000
(1) × (2)	0.002408	1	0.002408	0.1507	0.705241
Error	0.175748	11	0.015977		
Total sum	5.246240	14			

Water absorption					
Factors	Sum of squares	Degree of freedom	Mean square	F-calculated	*p*-value
(1) MRA/soil	22.17780	1	22.17780	12.4323	0.000034
(2) Cement	2.90405	1	2.90405	15.7699	0.007357
(1) × (2)	0.06480	1	0.06480	0.3519	0.574712
Error	1.10491	6	0.18415		
Total sum	26.25156	9			

Figure 6 shows the response surface obtained for the simple compression tests, as a function of the independent variables: cement content (%) on the vertical axis and the MRA/soil ratio on the horizontal axis. The response analyzed is the compressive strength (MPa), represented by the coloration of the surface in the *z* plane.

**Figure 6:**
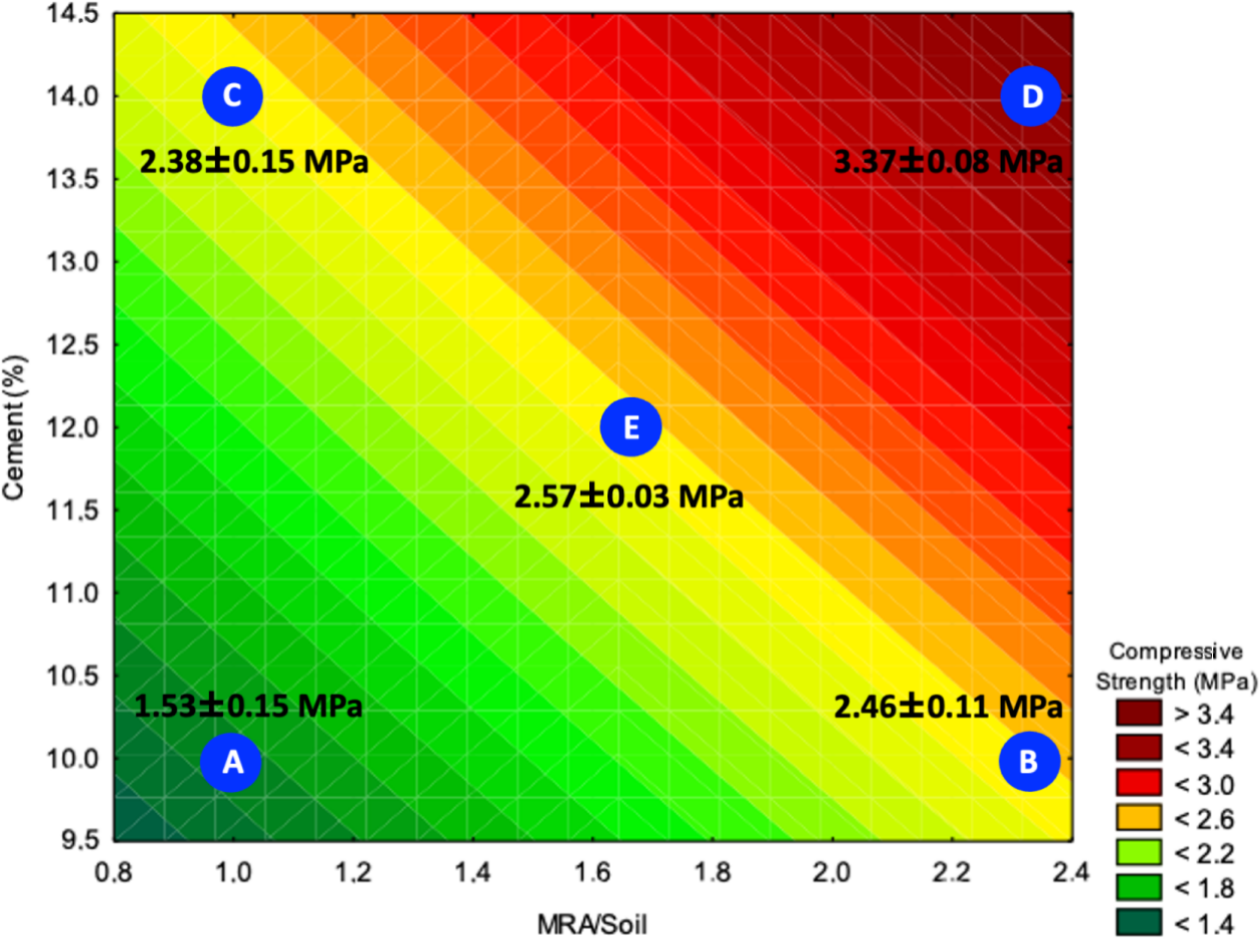
Response surface for compressive strength. The blue dots represent the samples studied and coded according to Table 3. The compressive strength values for these samples are shown in the figure.

In general, it can be observed that compressive strength increases with the simultaneous increase in the cement content and MRA/soil fraction, especially in the upper regions of the surface (red area). Point D, located in the condition of the highest cement content (14%) and highest MRA/soil ratio (2.33), presented the highest strength value, with 3.37 ± 0.08 MPa, showing that both factors act synergistically to reinforce the soil-cement matrix.

On the other hand, point A, with the lowest cement content (10%) and lowest MRA/soil ratio (1.00), showed the lowest compressive strength, with 1.53 ± 0.15 MPa. This indicates that, in isolation, low cement contents and little addition of granular material are not sufficient to ensure adequate mechanical performance of the soil-cement, corroborating previous studies that point to the need for a minimum of cement and granulometric correction for effective stabilization.^3,10^

Interestingly, the region with the lowest MRA/soil fraction associated with high cement contents (point C, 14% cement and 1.00 MRA/soil) resulted in intermediate strength (2.38 ± 0.15 MPa). In comparison, point B (10% cement and 2.33 MRA/soil) reached 2.46 ± 0.11 MPa. These results suggest that neither factor alone is sufficient to maximize strength, with the combined effect of the two being more relevant for formulation optimization.

The central point (E), representing the average experimental condition (12% cement and 1.67 MRA/soil), presented an intermediate strength of 2.57 ± 0.03 MPa, validating the response surface model and confirming the trend observed in the color gradient.

Table 9 shows the ANOVA of water absorption and indicates that the cement and MRA/soil factors are significant (*p*-value <5%), which means that water absorption is not the same at different levels. The interaction between the variables had no statistically significant influence ( *p* -value > 5%) on water absorption. The regression model’s determination coefficient (*R* ^2^) was estimated at 0.95791, indicating that approximately 95.79% of the variability in *z* is explained by the variability in *x* and *y*. Other factors not covered in this research can contribute only 4.21% of the variability in water absorption.

Figure 7 shows the response surface of water absorption (%) as a function of the cement content (vertical axis) and MRA/soil ratio (horizontal axis). This analysis aims to understand how these variables influence the apparent porosity of the material, which is directly related to the water absorption capacity of soil-cement specimens.

**Figure 7:**
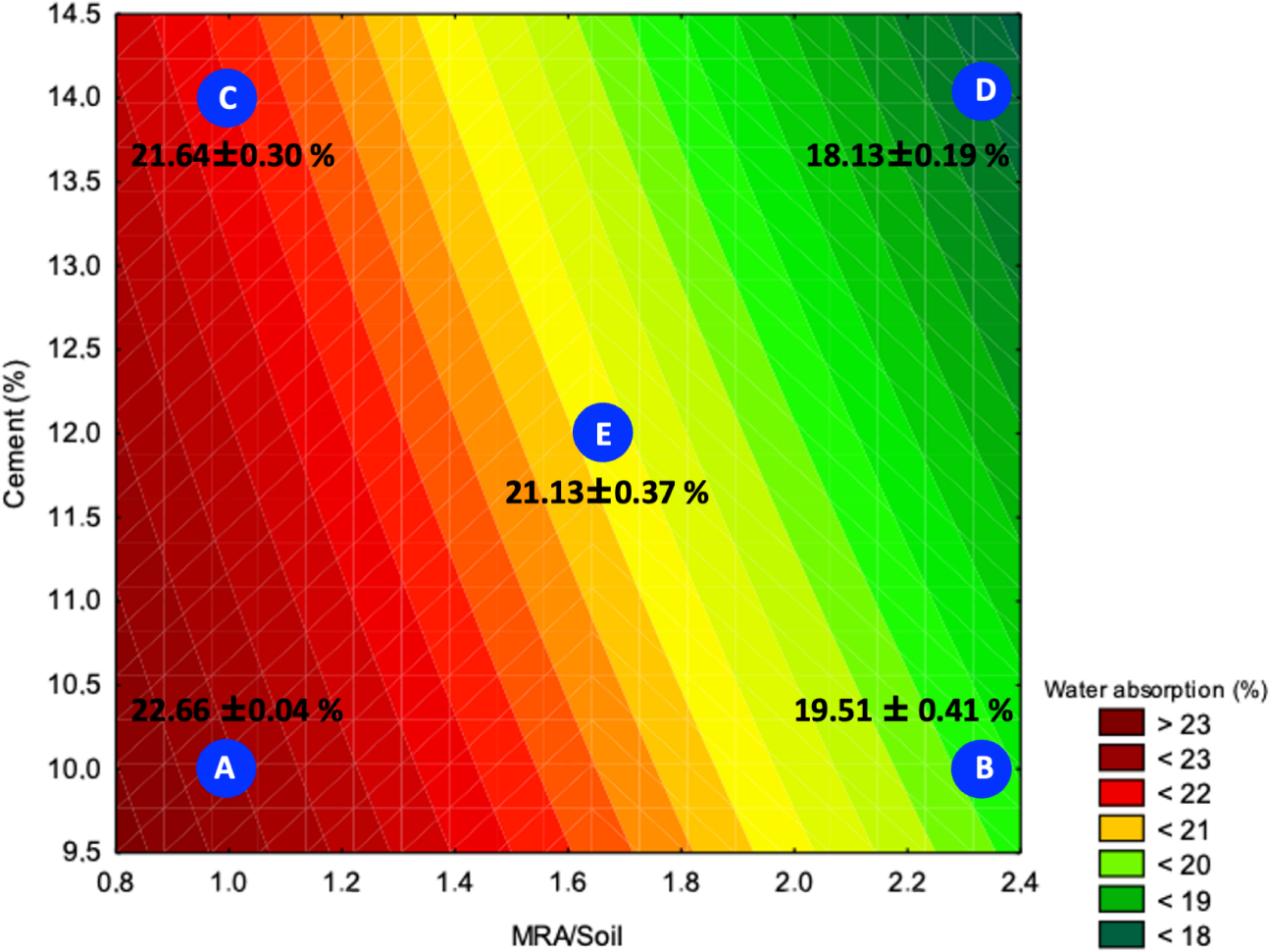
Response surface for water absorption. The blue dots represent the samples studied and coded according to Table 3. The water absorption values for these samples are shown in the figure.

The results show that increases in the MRA/soil ratio and cement content contribute to a reduced water absorption. The lowest absorption was recorded at point D (14% cement and 2.33 MRA/soil), with 18.13 ± 0.19%, indicating that conditions richer in granular material and binder favor matrix densification and void filling, resulting in lower permeability. This behavior suggests that higher cement contents reduce the blocks’ capillarity and that adding MRA improves compaction and reduces the formation of micropores.

On the other hand, the highest absorption was observed at point A (10% cement and 1.00 MRA/soil), with 22.66 ± 0.40%, representing the formulation with the lowest proportion of binder and granulometric correction. This shows that the absence of sufficient cement and the low presence of the sandy fraction compromise compaction, resulting in a greater number of interconnected pores and, consequently, greater water absorption.

Point C, also with low MRA/soil content (1.00) but high cement content (14%), showed intermediate absorption (21.64 ± 0.30%), indicating that cement alone is not capable of ensuring low absorption if there is insufficient granulometric correction to contribute to particle packing. Similarly, point B, with high MRA/soil (2.33) and low cement content (10%), performed better than point C, with 19.51 ± 0.41%, reinforcing the role of the granular fraction in reducing effective porosity.

Point E, located at the center of the experimental design (12% cement and 1.67 MRA/soil), showed intermediate absorption of 21.13 ± 0.37%, confirming the trend of gradual reduction in absorption with the increase of both factors.

These results are consistent with the findings of Ferrari et al.,^10^ who reported increased water absorption in blocks with cement replacement by ash. Although the present research does not directly address substitution by pozzolanic additions, the underlying logic remains that reductions in cement content or particle packing efficiency lead to higher porosity and absorption.

Soil-cement is a very flexible material that can be used in a variety of ways, such as in the construction of sidewalks in both urban areas and on highways, as well as being used in bags to reduce erosion caused by water on slopes and protect water outlets in canals, among other applications. It can also be used in constructing continuous walls or manufacturing blocks or bricks for masonry construction.^18^ It, therefore, becomes a suitable and viable alternative for the construction sector, adding value to the solid waste generated in the industry itself, given that in Brazil, it is estimated that around 45 million tons of construction and demolition waste will be generated in 2022.^36^

The dosage of 63% MRA, 27% soil (MRA/soil of 2.33), and 10% cement, considering the total mass percentage of solids in the composition, was the one that showed the most satisfactory results among the dosages evaluated given the valorization of solid waste from the reintroduction of CDW into the production system with greater incorporation of MRA, lower cement consumption, and the characteristics of compressive strength and water absorption.

From the response surfaces presented in Figures 5 and 6, it is possible to obtain the multiple regression models presented in eqs[Disp-formula eq1] and 2, which describe how the configuration of the MRA/soil (*x*) and cement (*y*) factors produce a response of compressive strength and water absorption in the soil-cement blocks, respectively.

1 Compressive strength(MPa)=−1.1657+0.5902x+0.20268y+0.01065xy

2 Water absorption(%)=27.0437−1.6917x−0.1886y−0.0677xy

Solving eq 1 using Microsoft Excel’s Solver add-in using the generalized reduced gradient (GRG) nonlinear algorithm,^37^ it is possible to predict the values of *x* and *y* considering the minimum compressive strength of 2.1 MPa, and the limits of the variables *x* and *y* evaluated when obtaining the multiple regression. The result for the MRA/soil ratio (*x*) is 1.71, which means a mixture of 63.10% MRA and 36.90% soil, and for the cement factor (*y*), a value of 10.22% was found. The predictability results are promising, as they allow for greater incorporation of CDW in manufacturing value-added products to contribute to recycling construction and demolition waste. In addition, predicting the need for a percentage of cement closer to the lower limit analyzed, 10.22%, indicates a cost reduction in producing soil-cement blocks.

The values obtained for the *x* and *y* factors were inserted into eq 2 to predict the water absorption indicated by the regression. A result of 21.04% was found, which aligns with the maximum water absorption predicted by the standard of 22%.

Ahmed Raza et al.^2^ report that excavated soil is part of the CDW, and a significant proportion of excavated soil needs to be used. Therefore, the production of different materials based on soil-cement is an alternative for reducing the environmental impacts caused by construction, which can result in a reduction in CO_2_ emissions, sustainable use of soil and CDW, and the saving of natural resources.

Using CDW in the form of MRA to achieve the ideal granulometry for clay soils used in the production of soil-cement blocks can be more economical than natural aggregates, especially in urban areas where waste is abundant and it also reduces landfill waste and promotes recycling, contributing to sustainable construction practices. The characteristics of the material produced allow for better retention and distribution of moisture, leading to better compaction and reduced plasticity, with improved mechanical strength, permeability, and durability.^38^

While achieving the ideal particle size is essential for optimizing the properties of clay soils with CDW, it is also important to consider the potential benefits of using natural aggregates and alternative additives. These alternatives offer unique advantages in specific contexts, such as sustainability and cost-effectiveness.^3,4,11,38^

## 4 Conclusions

The study evaluated the correction of clay soil with construction and demolition waste (CDW) for soil-cement production. The composition with the highest proportion of CDW showed satisfactory results in terms of compressive strength and water absorption as well as higher cement dosages. Correcting the soil to reduce the clay content and achieve the appropriate classification for use in soil-cement was necessary. Different cement contents and CDW/soil proportions were evaluated, and only one dosage failed to meet the reference values for compressive strength and water absorption. A decrease in optimum moisture content and an increase in maximum dry bulk density were observed as the soil-cement mix’s waste fraction increased. The composition with 63% MRA, 27% soil (MRA/soil of 2.33), and 10% cement showed satisfactory compressive strength and water absorption results for the lowest binder addition. The factorial experimental design indicated that the correction of clay soil with CDW, represented by the MRA/soil ratio factor, and the percentage of cement are statistically significant variables in the compressive strength and water absorption of soil-cement blocks, with an influence of more than 95.7% on the variability of the results. The results indicate that a granulometric correction is essential to make the analyzed soil suitable for soil-cement production. The definition of the ideal MRA ratio should consider not only granulometric suitability but also costs, material availability, and the desired mechanical performance for the final application. Therefore, using CDW to make soil-cement blocks meets national standards for mechanical performance, helping to mitigate the environmental impacts caused by the construction sector.

## Supplementary Material



## Notes

MRA characterization, Portland cement characterization, soil characterization: liquidity limit, plasticity index (PDF)

## Notes

J.F.B.: Conceptualization, Data curation, Formal analysis, Investigation, Methodology, Software, Writing – original draft C.A.d.L.: Data curation, Validation F.B.d.S.: Conceptualization, Data curation, Formal analysis, Investigation, Project administration, Resources, Software, Supervision, Validation, Writing – review and editing
