# Supplementary material for: Making Use of Construction Waste in Soil-Cement Mixtures: Granulometric Correction of Clay Soil
Source: ACS Omega. 2025 Aug 22;10(35):39471–81. doi: 10.1021/acsomega.4c10911 (PMC12423832; doi:10.1021/acsomega.4c10911)
Supplement: Supplementary file 1 [file ao4c10911_si_001.pdf]

Making Use of Construction Waste in Soil-Cement Mixtures: Granulometric Correction of Clay Soil

Jéssica Filipe Briskievicz[a], Carolina Angulski da Luz[b], Fernanda Batista de Souza[a,*]

[a]*Universidade Tecnológica Federal do Paraná (UTFPR), Câmpus Francisco Beltrão, Programa de Pós-graduação em Engenharia Ambiental: Análise e Tecnologia Ambiental (PPGEA), Rua Gelindo João Folador, n°2000, Francisco Beltrão, CEP 85602-863, PO Box 135, PR, Brazil*

[b]*Universidade Tecnológica Federal do Paraná (UTFPR), Câmpus Pato Branco, Departamento de Engenharia Civil, Via do Conhecimento, km 1, Pato Branco, CEP 85503-390, PR, Brazil*

* *Correspondence: fernandasouza@utfpr.edu.br; Tel.: +55-46-99941-7557 (F.B.S.)*

Supporting information

1. MRA characterization

The Brazilian Regulatory Standards (NBR) of the Brazilian Association of Technical Standards (ABNT), ABNT NBR NM 248:2003 method [1] was used to determine particle size. The ABNT NBR NM 46:2003 method [2] was used to determine the material passing through the 0.075 mm sieve, and the apparent specific mass was determined using the ABNT NBR 1211:2009 and ABNT NBR NM 27:2001 methods [3], [4].
Figure S1 shows the graph of the particle size analysis of the solid waste. According to the curve generated in the graph, the aggregate has a smooth, elongated horizontal distribution, indicating a well-graded continuous particle size, i.e., the aggregate has all the fractions in its particle size distribution curve, with 17% of the material passing through the 0.075mm sieve. The aggregate has a maximum characteristic dimension (MCD) of 2.36mm and a fineness modulus (MF) of 1.64. It is classified as fine sand because it has a fineness modulus of less than 2.4 [5].

Figure S1 - MRA particle size analysis graph

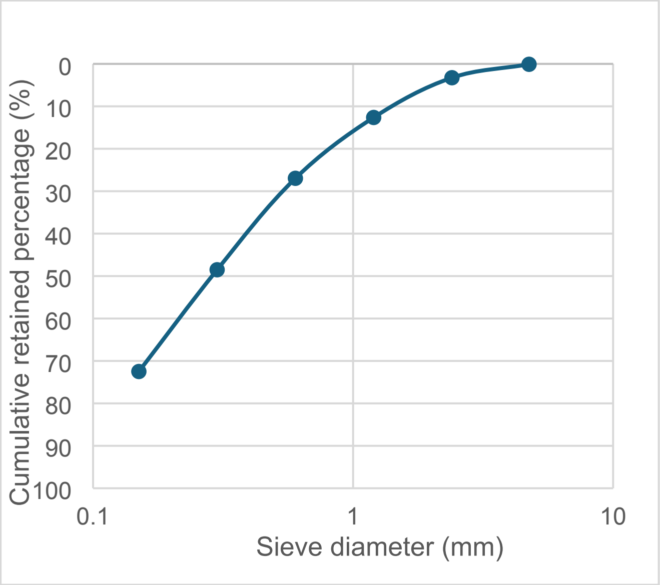

2. Portland cement characterization

Table S1 presents the technical specifications provided by the manufacturer for Portland cement CP II-Z. According to the manufacturer, the average specific mass is 2.92 g/cm³; this cement has 6 to 14% pozzolan in its composition and requires curing by spraying water for at least 7 days.

Table S1 – Physicochemical characteristics of CP II – Z

**Química**		**Físicas**	
Parameters	(%)	Parameters	-
Al_2O_3	8.58	Hot expandability (mm)	0.25
SiO_2	21.25	Setting Time (min)	Start 255<br>End 310
Fe_2O_3	3.23	Normal consistency (%)	28.5
CaO	52.74	Blaine (cm²/g)	3.743
MgO	2.69	Sieve 200 (%)	1.27
SO_3	2.55	Sieve 325 (%)	6.38
Fire Loss	7.12	Compressive strength (MPa)	1 day – 14.4
Free CaO	0.87		3 days – 28.1
Insoluble Waste	10.92		7 days – 34.4
Alkaline equivalent	0.77		28 days – 42.6

3. Soil characterization

3.1. Liquidity Limit

The liquid limit (LL) test was guided by NBR 6459 [6], and the equipment shown in Figure S2 was used for its execution. This equipment is equipped with a concave plate and a crank that, when turned, causes a blow to the plate. With successive blows, a slit previously made in the sample placed on the concave plate closes. The number of blows required to close approximately 1 cm of the scratch and the moisture content of this point is used to prepare a graph, the line equation which provides the soil moisture for the liquid limit represented by 25 blows.
The procedure was repeated for different moisture contents in two replicates. Water was added to a soil sample and homogenized for 15 to 30 minutes. The moistened and homogenized sample was spread on the concave plate, a cut was made in the sample with

a chisel, as exemplified in Figure S3, and blows were applied with the apparatus crank. As soon as the lower edges of the cut closed to one centimeter, the number of blows used was noted, and a small sample was removed to determine the moisture content according to NBR 6457[7].

With the results of the number of blows required for each moisture content considering five moisture points starting from the driest point to the wettest, the standard indicates the range of 35 to 15 blows of the equipment, a graph with an adjusted straight line was created. The equation of the straight line determined the moisture content of the soil for the liquidity limit relative to 25 blows for each sample.

Figure S2 – Equipment and soil sample

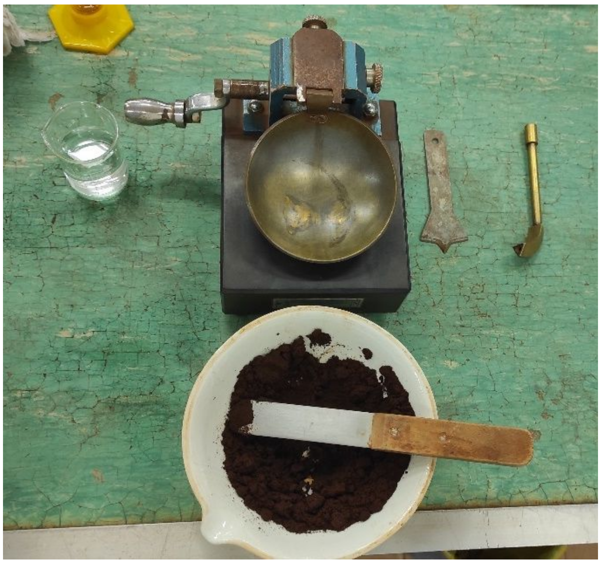

Source: Own authorship (2021)

Figure S3 - Sample with the cut for the Liquidity Limit test

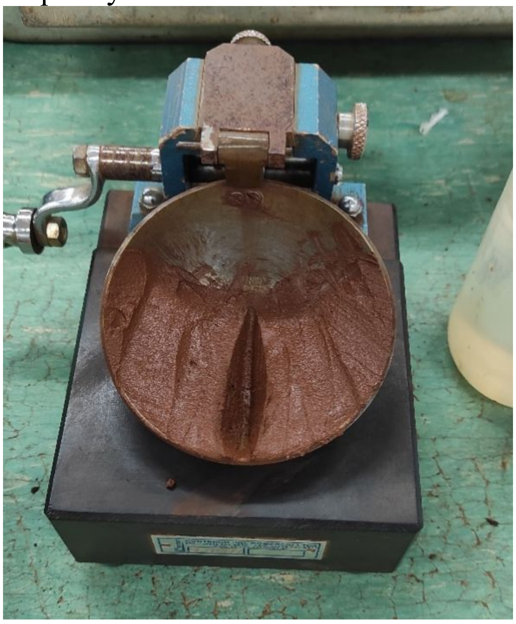

Source: Own authorship (2021)

Table S2 presents the results of the liquid limit test of the natural soil for the two replicates. Figure S4 refers to the graph of replicate 1 and Figure S5 to the graph of replicate 2. The results of the liquidity limit tests for the soil-cement mixtures studied are shown in Figures S6 to S11.

Table S2 - Liquidity limit of natural soil

	Number of blows	Wet mass – M1 (g)	Dry mass – M2 (g)	Tare mass – M3 (g)	Moisture content (%)
**Replicate 1**	35	26.74	25.09	22.21	**57.40**
	30	27.85	26.17	23.28	**58.27**
	28	29.66	26.94	22.35	**59.26**
	21	28.51	26.27	22.63	**61.54**
	16	28.27	26.27	23.11	**63.35**
**Replicate 2**	40	25.49	24.43	22.46	**53.67**
	35	28.29	27.43	25.85	**54.53**
	30	27.07	25.37	22.33	**55.83**
	23	25.83	24.47	22.12	**57.87**
	15	26.89	25.09	22.14	**60.98**

Figure S4 - Soil liquid limit test graph: replicate 1

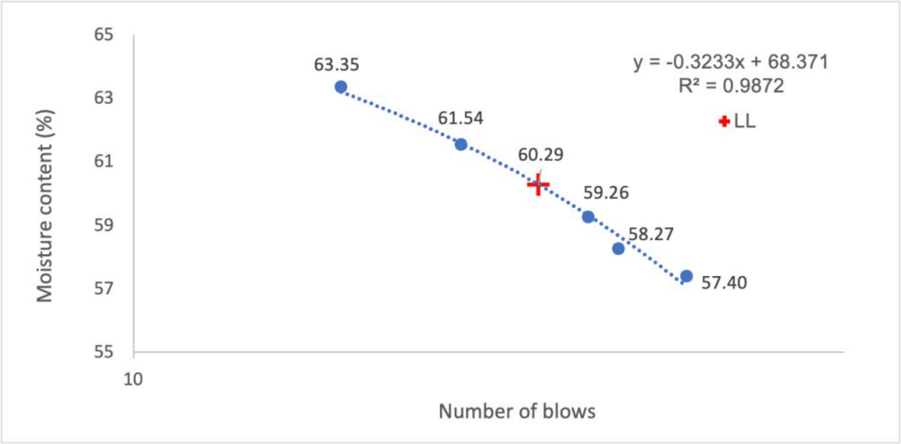

Figure S5 - Soil liquid limit test graph: replicate 2

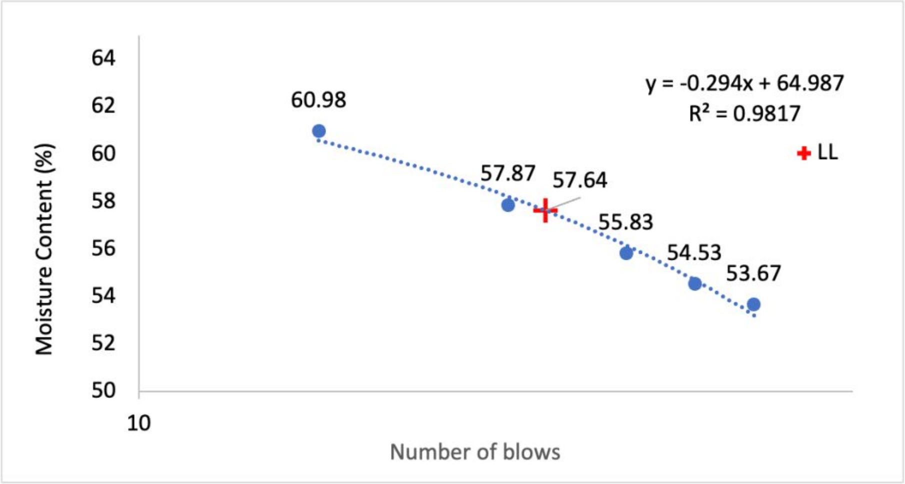

Figure S6 - Graph of the LL test of the 50/50 corrected soil: replicate 1

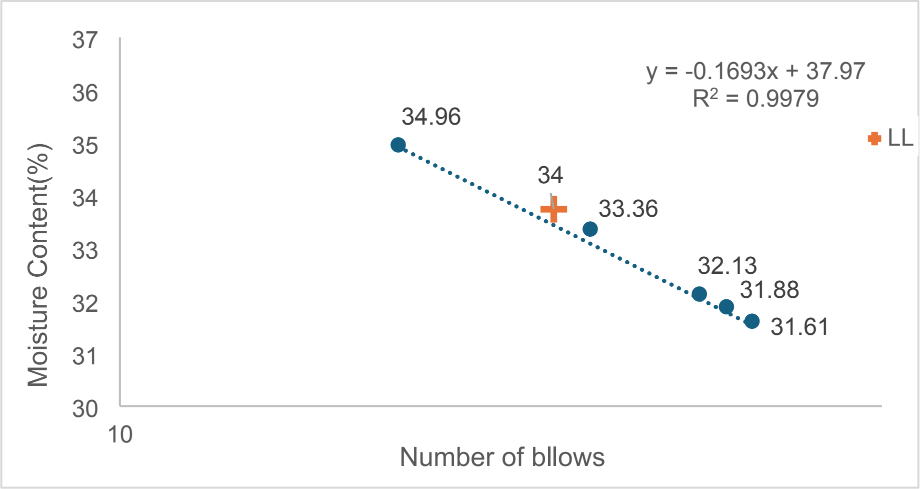

Figure S7 - Graph of the LL test of the 50/50 corrected soil: replicate 2

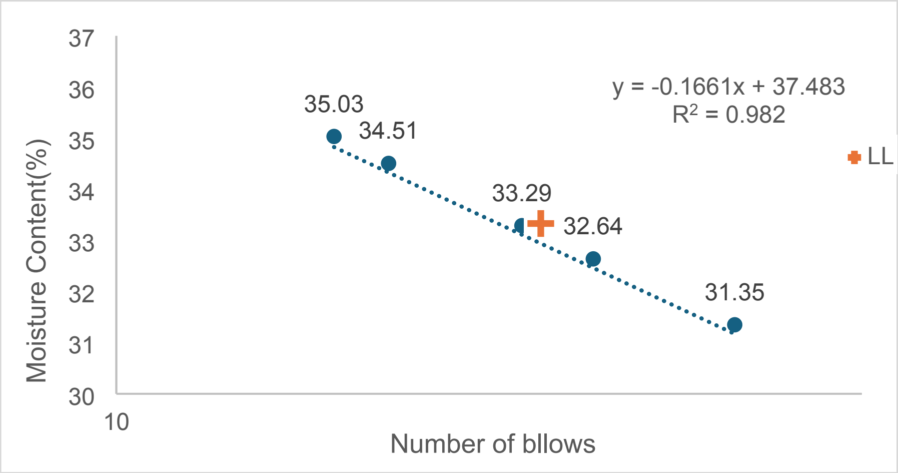

Figure S8 - Graph of the LL test of the 32.5/67.5 corrected soil: replicate 1

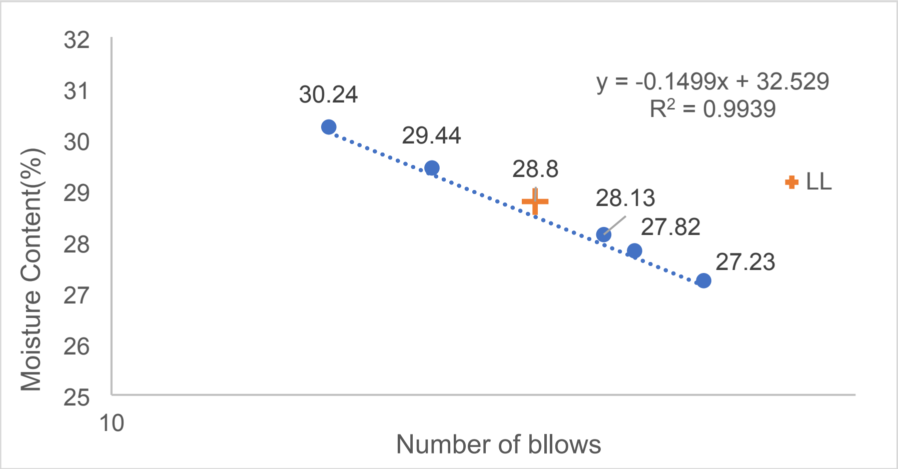

Figure S9 - Graph of the LL test of the 32.5/67.5 corrected soil: replicate 2

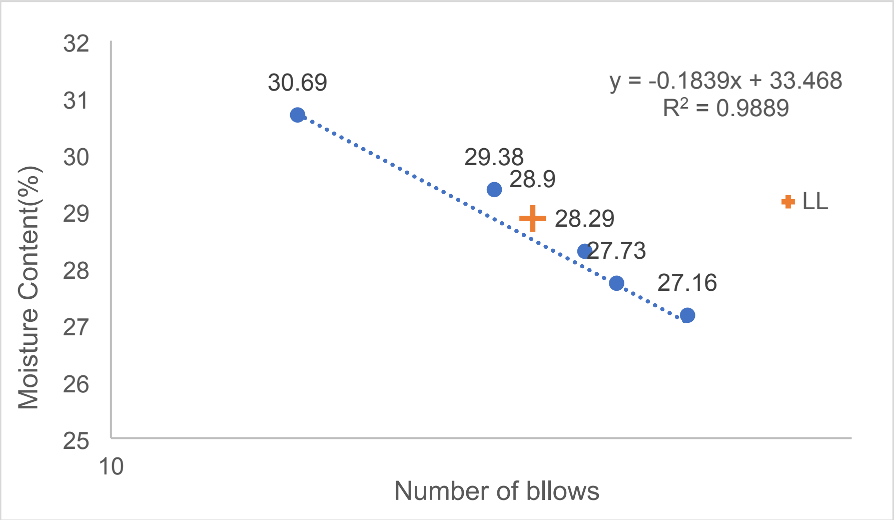

Figure S10 - Graph of the LL test of the 30/70 corrected soil: replicate 1

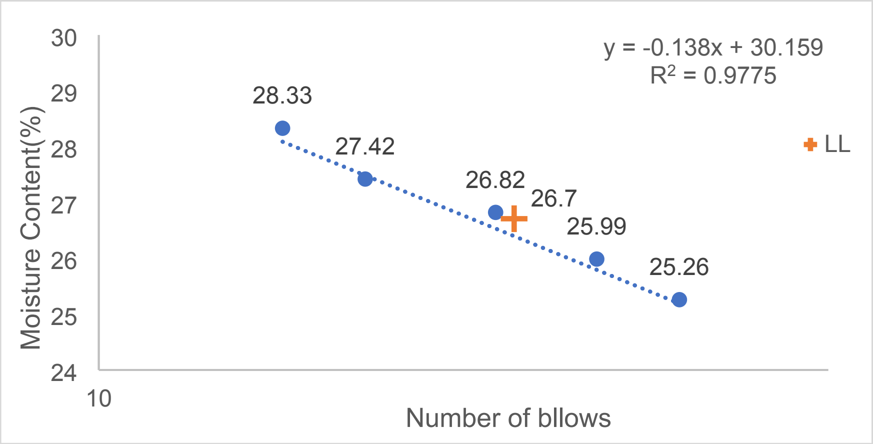

Figure S11 - Graph of the LL test of the 30/70 corrected soil: replicate 2

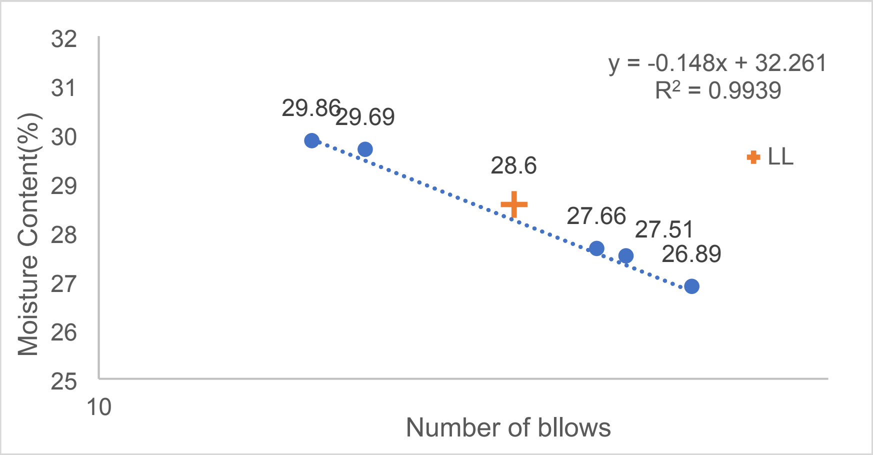

3.2. Plasticity index

The plasticity index (PI) test was carried out by NBR 7180 [8] and repeated three times for each sample. A soil sample was placed in a porcelain capsule, then distilled water was added and the sample was mixed vigorously for around 20 minutes. When the sample had the consistency of a plastic paste, 10g of the material was removed, and a small ball was molded, as shown in Figure S12a. The ball was placed on a glass plate and pressed with the palm until it formed a cylinder, as shown in Figure S12b. When the cylinder reached a diameter of 3 mm and a size of 100 mm with small cracks, it was immediately transferred to a metal capsule, and the moisture content was determined by NBR 6457 [7]. Table S3 shows the plasticity limit of natural soil, and Tables S4 to S6 show the results of the soil-cement mixtures studied.

Figure S12 – Plasticity limit test: a) ball-shaped molding, b) cylinder molding

a)

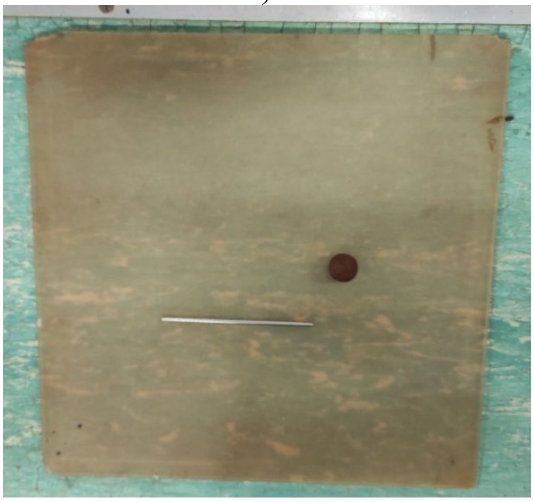

Source: Own authorship (2021)

b)

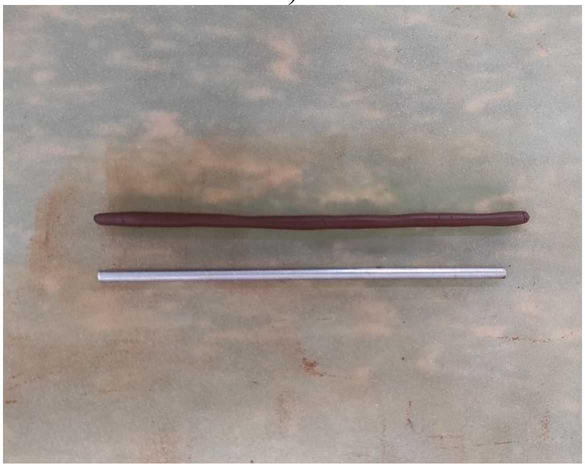

Source: Own authorship (2021)

Table S3 - Plasticity limit of natural soil

	Wet mass – M1 (g)	Dry mass – M2 (g)	Tare mass – M3 (g)	Moisture content (%)
	24.15	23.59	22.26	**42.11**
**Replicate 1**	23.85	23.31	22.03	**42.19**
	26.98	26.48	25.24	**40.32**
	24.29	23.84	22.75	**41.38**
**Replicate 2**	22.59	22.08	20.92	**43.97**
	23.62	23.12	21.92	**41.67**

Table S4- Plasticity limit of corrected soil 50/50

	Wet mass – M1 (g)	Dry mass – M2 (g)	Tare mass – M3 (g)	Moisture content (%)
	12.31	11.96	10.64	**26.52**
**Replicate 1**	12.91	12.53	11.06	**25.85**
	12.26	11.91	10.50	**24.47**
	23.07	22.80	21.53	**20.87**
**Replicate 2**	24.35	24.10	23.13	**25.77**
	23.60	23.30	22.04	**23.81**

Table S5 - Plasticity limit of corrected soil 32.5/67.5

	Wet mass – M1 (g)	Dry mass – M2 (g)	Tare mass – M3 (g)	Moisture content (%)
**Replicate 1**	22.89	22.51	20.97	**24.68**
	24.04	23.65	22.09	**25.00**
	23.85	23.54	22.28	**24.60**
**Replicate 2**	23.61	23.30	22.00	**23.80**
	22.90	22.60	21.41	**25.21**
	23.35	23.00	21.64	**25.74**

Table S6 - Plasticity limit of corrected soil 30/70

	Wet mass – M1 (g)	Dry mass – M2 (g)	Tare mass – M3 (g)	Moisture content (%)
**Replicate 1**	23.82	23.50	22.26	**25.81**
	23.70	23.40	22.03	**21.97**
	23.75	23.48	22.36	**24.46**
**Replicate 2**	27.21	26.80	25.24	**26.35**
	24.81	24.50	23.24	**24.60**
	22.61	22.40	21.50	**23.33**

REFERENCES

[1] ABNT, “NBR NM 248: Agregados - Determinação da composição granulométrica,” 2003, *Associação Brasileira de Normas Técnicas, Rio de Janeiro*.

[2] ABNT, “NBR NM 46: Agregados - Determinação do material fino que passa através da peneira 0,075 mm,” 2003, *Associação Brasileira de Normas Técnicas, Rio de Janeiro*.

[3] ABNT, “NBR NM 27: Agregados - Redução da amostra de campo para ensaios de laboratório,” 2001, *Associação Brasileira de Normas Técnicas, Rio de Janeiro*.

[4] ABNT, “NBR 7211: Agregados para concreto - Especificação,” 2009, *Associação Brasileira de Normas Técnicas, Rio de Janeiro*.

[5] L. A. F. Bauer, *Materiais de construção, v. 1*. Rio de Janeiro: LTC, 2019. [Online]. Available: https://research.ebsco.com/linkprocessor/plink?id=fb6039e5-4de9-3db1-8f73-950d3cb277ce

[6] ABNT, “NBR 6459: Determinação do limite de liquidez,” 2016, *Associação Brasileira de Normas Técnicas, Rio de Janeiro*.

[7] ABNT, “NBR 6457: Solos — Preparação de amostras para ensaios de compactação, caracterização e determinação do teor de umidade,” 2024, *Associação Brasileira de Normas Técnicas, Rio de Janeiro*.

[8] ABNT, “NBR 7180: Solo — Determinação do limite de plasticidade,” 2016, *Associação Brasileira de Normas Técnicas, Rio de Janeiro*.
